# The CDC crossmatch in the era of flow cytometric cross-match and single antigen beads

**DOI:** 10.1590/2175-8239-JBN-2021-0110

**Published:** 2021-07-02

**Authors:** Grace Kao Mahowald

**Affiliations:** 1 Massachusetts General Hospital, Department of Pathology, Boston, MA, USA.; 2 Harvard Medical School, Boston, MA, USA.

The complement-dependent cytotoxicity crossmatch (CDC-XM), a technique that uses T and B lymphocytes to detect donor-specific antibodies (DSA) via activation of the classical pathway of complement, has been widely used for the detection of alloantibodies in transplant patients since it was introduced in the 1960s. In their seminal paper published in 1969, Patel and Terasaki demonstrated 24 out of 30 immediate graft failures in kidney transplants performed across a positive crossmatch, compared to eight out of 195 transplanted across a negative crossmatch, and concluded that “the ethics of transplanting kidneys without the prior knowledge of the results of the crossmatch test…can reasonably be expected to be questioned in the face of this evidence”^1^. As a result of this study, prospective crossmatching became part of routine clinical practice for kidney transplantation.

Although the CDC-XM revolutionized the practice of kidney transplantation, the current need for routine CDC-XM is less clear with the advent of more sensitive techniques. The addition of anti-human globulin (AHG) to the CDC-XM increased the sensitivity compared to the standard CDC-XM, but the assay remained relatively insensitive. The flow cytometric crossmatch (FCXM), introduced in 1983, allowed for increased sensitivity in detecting IgG from recipient serum bound to the surface of T and B lymphocytes through the use of a fluorescently-labeled F(ab’)_2_ anti-human IgG^2^. The FCXM has undergone modifications with three-color analysis and, more recently, a rapid optimized protocol that incorporates a 96-well plate format and other time savings (the Halifax and Halifaster protocols)^3^. The subsequent development of solid-phase HLA antibody screening techniques, including Luminex single antigen beads (SAB), has greatly increased the sensitivity of antibody detection and, in conjunction with the donor HLA type, allows for a virtual assessment (or virtual crossmatch, if the patient proceeds to transplant without a prospective physical crossmatch) that can identify DSA at levels that are below the limit of detection of FCXM.

With improved sensitivity of crossmatching has come improvement in clinical outcomes. A study of patients undergoing kidney re-transplantation with deceased donors showed that the seven-year death-censored graft survival in patients with negative T-cell FCXM (68%, n=106) was comparable to that of patients receiving their first kidney transplant (72%, n=889), and significantly better than that of patients in whom only the AHG CDC-XM was used (45%, n=174)^4^. A large multi-center trial of incompatible live donor kidney transplantation comparing CDC+, FCXM+/CDC-, DSA+/FCXM-, and DSA- patients found that graft loss essentially mirrored each of these risk categories. There was a significant increase in graft loss in the first year post-transplant in CDC+ and FCXM+/CDC- patients compared to DSA- patients, with an adjusted hazard ratio of 5.01 and 1.64, respectively^5^. A meta-analysis of 1119 kidney transplant patients with negative CDC and FCXM showed that the presence of DSA detected by solid-phase assays is associated with a significantly increased risk of antibody-mediated rejection (ABMR) and graft failure (relative risk 1.98 and 1.76, respectively)^6^, supporting the clinical relevance of DSAs detected only by solid-phase assays.

In this issue, Abud et al. (2021)^7^ compare a cohort of patients with pre-transplant assessment performed by CDC-XM only to a subsequent cohort transplanted after assessment by FCXM only^7^. There was no difference in the one-year patient survival or graft survival between the two groups, although 2/15 graft losses were due to immunological causes in the CDC-XM group, compared to 0/15 in the FXCM group, and 3/68 patients in the CDC-XM group had acute ABMR on biopsy, compared to 0/63 in the FCXM group. Of note, all patients in both groups were screened for the presence of DSA by SAB within four months prior to transplantation, and were overall similar in terms of presence of pre-transplant DSA and sum of MFIs of DSAs. The three patients in the CDC-XM group that developed acute ABMR were noted to be DSA-negative at the time of transplant. In this study, the lack of difference between the CDC-XM group and FCXM group is unsurprising, given that Luminex SAB testing was used for DSA detection in both groups. Since the method used for DSA detection is more sensitive than either of the crossmatch techniques studied, patients with moderate to high level DSA were presumably not transplanted on this basis, rather than the result of the crossmatch per se.

With the routine use of sensitive solid-phase assays, the CDC-XM and even FCXM arguably become of secondary importance in the pre-transplant assessment of donor-recipient compatibility. However, there are situations in which a physical crossmatch is still informative, such as in cases with allele-specific antibodies or antibodies specific for particular DQA1/DQB1 combinations, suspected false-positive SAB reactivity (antibody to denatured antigen), or antibody to an epitope shared across multiple beads. In these situations, a prospective flow crossmatch can provide insight into the significance of the DSA, while the CDC-XM is generally not sensitive enough to give the answers that are needed. It is important, though, to keep in mind that both FCXM and CDC-XM can also be positive in the absence of HLA DSA. Some false-positive CDC-XMs are attributable to IgM antibodies and can be resolved through serum treatment with dithiothreitol or heat. However, other patients, particularly those with autoimmune diseases, may have persistent false positive CDC-XM and/or FCXM. In addition, therapies such as rituximab interfere with both varieties of B-cell crossmatch. There is no perfect assay, but having the ability to assess DSA by orthogonal methods in these complex situations can be invaluable.

## Abbreviations

CDC-XM: - complement-dependent cytotoxicity crossmatch
AHG: - anti-human globulin
FCXM: - flow cytometric crossmatch
SAB: - single antigen beads
DSA: - donor specific antibody
ABMR: - antibody mediated rejection
